# Clustering-Based Ensemble Learning for Activity Recognition in Smart Homes

**DOI:** 10.3390/s140712285

**Published:** 2014-07-10

**Authors:** Anna Jurek, Chris Nugent, Yaxin Bi, Shengli Wu

**Affiliations:** School of Computing and Mathematics, University of Ulster, Jordanstown, Shore Road, Newtownabbey, Co. Antrim BT37 0QB, UK; E-Mails: cd.nugent@ulster.ac.uk (C.N.); y.bi@ulster.ac.uk (Y.B.); s.wu1@ulster.ac.uk (S.W.)

**Keywords:** activity recognition, classifier ensembles, clustering, smart homes

## Abstract

Application of sensor-based technology within activity monitoring systems is becoming a popular technique within the smart environment paradigm. Nevertheless, the use of such an approach generates complex constructs of data, which subsequently requires the use of intricate activity recognition techniques to automatically infer the underlying activity. This paper explores a cluster-based ensemble method as a new solution for the purposes of activity recognition within smart environments. With this approach activities are modelled as collections of clusters built on different subsets of features. A classification process is performed by assigning a new instance to its closest cluster from each collection. Two different sensor data representations have been investigated, namely numeric and binary. Following the evaluation of the proposed methodology it has been demonstrated that the cluster-based ensemble method can be successfully applied as a viable option for activity recognition. Results following exposure to data collected from a range of activities indicated that the ensemble method had the ability to perform with accuracies of 94.2% and 97.5% for numeric and binary data, respectively. These results outperformed a range of single classifiers considered as benchmarks.

## 1. Introduction

Monitoring patients' activities of daily living (ADL) is a popular approach to assessing physical and cognitive well-being remotely from a person's home environment. ADLs refer to activities performed on a daily basis, usually within the home environment, such as toileting, grooming, cooking or undertaking light housework. Smart Homes are residences equipped with different types of sensors that can monitor such activities. The sensors have the ability to record a person's interaction within the environment itself, for example, recording whenever a cupboard is open or closed or the turning on or off of a domestic appliance [1,2]. From a data analysis perspective it is therefore possible to infer from the change of a sensor's state that a person in the environment has interacted with a specific object. Based on the interactions captured it is possible to detect the change of state associated with an object/region within the environment. This type of monitoring system can provide information that can be used to infer certain elements of health and behavioural status.

In its basic form the output from a sensorised environment is a stream of sensor activations, which have occurred over a period of time. Analysis of the data can lead to the recognition of the activities, which have been performed within the environment. Such a stream of sensor data needs to be initially partitioned into segments, which represent single activities [3]. Each activity can be viewed as being composed of a combination of actions, such as taking a cup from a cupboard or pouring water from a kettle and can be represented by many different combinations of individual actions. For example, making tea may involve taking milk from the fridge or it may involve preparing the drink with no form of flavourings. In addition, different activities may last for different durations of time, for example sleeping or using the telephone. Activities themselves can be considered to have different levels of complexity. Cooking dinner, for example, involves a larger number of actions compared to preparing a cup of tea.

Following data segmentation, the next task involves recognising, which from a set of predefined activities has been represented by a given segmented stream of actions. This problem is referred to as activity recognition and it can be considered as a classification process of an instance representing a string of sensor activations into one from a possible number of classes representing activities such as cooking dinner or preparing a drink.

The focus of the study presented in this paper has been directed towards the design and evaluation of a previously developed Cluster-Based Classifier Ensemble (CBCE) method [4] within the realms of an activity recognition problem within a smart environment. The preliminary results of this work were presented in [5]. The CBCE method is compared with a number of single classification models for benchmarking purposes. Two types of sensor data representation are considered, namely numeric and binary. An analysis is undertaken in an effort to investigate which is the best form of data representation for the ensemble method. The paper is organised as follows: Section 2 presents related work in the area of activity recognition and ensemble learning. Section 3 describes in detail the CBCE method including the training and classification process. In Section 4 we demonstrate the data pre-processing process that has been undertaken followed by a case study presented in Section 5. In Section 6 we present and discuss the experimental results following exposure of the method to a dataset collected from a smart environment. Section 7 concludes the paper with Conclusions and Future Work.

## 2. Related Work

### 2.1. Sensor-Based Activity Recognition

The problem of activity recognition based on processing data obtained through low-level sensors, has been investigated in many studies [6]. Different approaches that have been explored can be roughly categorised as being either of a data-driven or knowledge-driven approach.

In the data driven categorisation, the most popular techniques which have been considered are classification models based on probabilistic reasoning for example Naïve Bayes (NB) classifiers [7], Dynamic Bayesian Networks [8], Hidden Markov Models (HMMs) [9], and Conditional Random Fields (CRF) [10]. Other algorithms such as Decision Trees [11] or Artificial Neural Networks (ANN) [12] have also been evaluated in activity recognition problems. In the aforementioned studies these machine-learning methods have been presented as being successful in the process of activity recognition. In addition, probabilistic activity recognition algorithms have been reported as having the advantage of handling noise and incomplete and uncertain sensor data [13]. There are, however, a number of limitations that may cause a range of application difficulties. Learning-based methods require a large number of training examples to provide good levels of generalisation. Within the application domain of smart environments access to a large validated data set can be viewed as being difficult. Beside this, probabilistic methods that require finding joint probability distributions and assume independence among observations for example NB or HMM, have difficulty in representing concurrent or interwoven activities [14]. CRF [14] requires finding conditional, instead of joint probability and allows for non-independent and arbitrary relations to be made among the observations, subsequently making it more flexible. HMM and CRF, however, have limitations related to sequential representation of activities which cause problems whilst modelling more complex activities with interleaved or concurrent sub-activities. In addition, probabilistic models do not deal very well with the problem of multiple representations of a single activity.

Some work has been undertaken in an effort to investigate how ensemble methods can be applied with the aforementioned techniques in activity recognition [15]. Three base classifiers were considered, namely NB, HMM and CRF. Boosted DT was applied as the combining method. As an input for the final classification model the probability distributions obtained from the base classifiers together with the activity feature values were considered. It was hypothesised that such an approach can provide a generalised model for common activities that can be applied for different resident types in multiple environment settings. Eleven data sets were considered in the experiments. For the experimental evaluation the leave-one-out technique was applied for base classifiers and for the ensemble method. The model was first trained with ten data sets and then tested with the left-out data set. This facilitated an investigation relating to how the model performed if no specific data to the environment were provided during training. The ensemble method outperformed base classifiers in 10 data sets. This indicated that the classifier ensemble approach is more suitable than a single classifier for training general activity models on data which is different than that used for testing.

In [16] authors evaluated three different representations of binary sensor data previously introduced in [10]; “Raw”, “ChangePoint” and “LastSensor”. The “Raw” technique indicated which of the sensors were active within a time interval. With “ChangePoint” data was recorded when a binary sensor changed its value, from zero to one or from one to zero. The “LastSensor” representation indicated which sensor was active, as the last one. Two hybrid systems were proposed for recognition of ADLs from smart home environments based on these concepts. Both systems were created by combinations of HMM with discriminative classifiers, namely ANN and support vector machines (SVM). The two models together with a number of single classifiers were evaluated using each of the feature representations standalone and combined. Following experimentations it was demonstrated that the SVM/HMM approach obtained significantly better results than any of the other techniques. Beside this, it was found that in general the models performed the best while applied with “LastSensor” and combination of “ChangePoint” + “LastSensor” feature representations.

A new approach to ADL recognition, based on Evolving Fuzzy Systems, was proposed in [17]. With this approach a number of fuzzy rules were composed for each activity. The rules described the most significant features for the classification process of each of the activities and were determined based on an available training set. Two evolving classifiers were developed and following empirical evaluation it was demonstrated to perform equally well with a HMM based approach. Computational efficiency and ability to adapt automatically to the changes occurring in ADL performance are considered as advantages of the proposed models.

Two commonly used knowledge-driven approaches applied in activity recognition are based on logical modelling or evidential theory. In a logical modelling based approach [18] logical knowledge and logical reasoning is applied to recognise an activity. In an evidential theory based approach [19,20] sensor readings are considered as evidence of occurrence of an activity. They are subsequently combined to determine the belief that one of the activities took place. Given that the knowledge-driven approaches incorporate knowledge, they do not require a large amount of data for training. In addition to this, the knowledge can be used in multiple systems. To a certain extent this makes them more appealing for use with real life applications than probabilistic models. In addition, similar to learning-based methods, they can handle noise and incomplete sensor data. There is, however, a negative side to this approach that is related to the knowledge that needs to be provided for the model. It may be difficult to define specifications of activities by applying only expert knowledge, which is not subsequently deemed to be subjective.

### 2.2. Ensemble Learning for Classification

A combination of multiple classifiers, often referred to as a classifier ensemble, is a group of classification models whose individual decisions are merged in some manner to provide, as an output, a consensus decision. The overarching aim of this approach is to combine outputs of a number of classification models, also referred to as base classifiers, to generate a single aggregated output that outperforms any of the base classifiers operating in isolation. Constructing a classifier ensemble can be considered as a two-step process. In the first step a collection of different base classifiers is created [21,22]. In the second step, a combination model is created for the purpose of merging the outputs of the base classifiers to obtain the final decision [23–25]. Combining multiple classifiers has been presented by many studies as an effective way of improving classification accuracy of a single classifier [25–27]. The most popular ensemble learning based classification techniques are referred to as bagging, boosting and stacking. With bagging the training sets are randomly chosen *k* times with replacement (bootstrap techniques) from the original data set [28]. It is possible that some instances may appear more than once in some of the training sets and some instances may not be present at all. As a result *k* training sets, with a size equal to the original set of data, are obtained. Following this, a single learning method is applied to train one classifier with each of the generated training sets. Consequently we obtain *k* different classifiers that make a prediction for an unseen instance. A voting technique is applied for the purpose of combining predictions of the base classifiers and making the final decision. An ensemble technique based on a similar approach to bagging is referred to as Random Forest (RF) [21]. The training algorithm for RF applies a bootstrap technique to select samples from the training set and fits trees to these samples. In addition, a random subset of features is applied at each candidate split in the learning process of the decision trees.

Boosting [29] is an iterative approach where the distribution of the training set is dynamically altered, based on the classifier's accuracy. After each base classifier is built and added to the ensemble, all instances are re-weighted: those that have been correctly or incorrectly classified loose or gain weight in the decision making process, respectively. The modified distribution of the training set is considered in the training process of the next base classifier. The final prediction is made by taking a weighted vote of each base classifier's prediction. The weights are proportional to the accuracy of each classifier relative to its training set.

An alternative technique of combining multiple classifiers is based on the meta-learning approach and is generally referred to as stacking [30]. It is usually applied to combine models that were built using different learning methods on a single data set. Within the process of stacking a collection of base-level classifiers is initially generated (level-0). Following this, all instances from the validation set are classified by all base classifiers. The results of this process compose a training data set for a meta-model. In the following step, a meta-model is built for combining the decisions of the base-level models. A new test pattern is firstly classified by all base-level models. Based on these predictions, the meta-classifier makes a final decision.

It has been demonstrated by many studies that an appropriately trained classifier ensemble can provide better results than a single classifier. By averaging the decisions of different experts we decrease the risk of selecting the incorrect classifier, from which a decision is to be made. Given different human behaviours, some representation of activities may be very confusing to interpret. For example, activities such as preparing dinner or breakfast may be represented by very similar sensor readings. Consequently, it is beneficial to obtain a range of different opinions rather than applying a single model. Beside this, two activities may happen at the same time, or they can be interleaved. Representation of such an event may be classified as one of the two activities, depending on the subset of sensors (features) that are considered whilst making the decision. It is therefore difficult to train a single classification model that will be able to accommodate all such cases. Generating a collection of classifiers by training them on different subsets of features may therefore offer a better solution.

The approach presented in this paper can be considered as a modified bagging technique. The findings from our previous work have shown [6] that CBCE performs better than a number of single and ensemble-based classification methods when considering a number of publicly available data sets. Despite the relatively high computational cost of the training phase, the proposed approach has been considered as being appealing from the classification point of view. In addition, the CBCE approach performed significantly better with very small data sets than the other methods considered in [6]. This suggests that CBCE does not require a large number of instances to train the final model making it suitable for real world applications.

Given the fact that CBCE is a cluster-based classification method, we hypothesise that it is more adaptable to the type of data applied in activity recognition problems. One of the issues in sensor-based activity recognition is related to the fact that the same activity can be performed in many different ways, hence making it difficult to define a general description for each activity [31]. With CBCE the activities are modelled as clusters hence it is not required to process each instance from the data set individually while training the classification model.

## 3. Cluster-Based Classifier Ensemble

With the CBCE method one base classifier is generated as a collection of clusters built on the training set with a different subset of features. Presentation of a new instance is assigned to its closest cluster from each collection. The final prediction is made based on the class labels of the instances that belong to the selected clusters. The ensemble method has been previously evaluated on open data sets from the machine learning domain, however, it has not been previously applied within the field of activity recognition.

### 3.1. Creating Base Classifiers

As an input to the process, one dataset *D* and a number *K* representing the size of the ensemble are provided. The value of the parameter *K* is determined in advance based on the performance of the final model with training data. In the presented study, following the evaluation of the model on the training set, the size of the ensemble was selected as 30. The clustering process is performed a number of times whilst varying two parameters, namely a subset of features applied while calculating the distance between 2 instances and the number of clusters generated in the clustering process. Consequently we obtain a set of different cluster collections that represent base classifiers. The training process of a single base classifier is performed in 3 steps. In Step 1, the subset of features {*f_k_*_1_,…,*f_k_*_*l*_} and the number of clusters *P_k_* are randomly selected. Index *k* refers to the classifier in the collection and *l* ≤ *L*, where *L* represents the total number of features. The number of selected features is randomly chosen as a value between 1 and the total number of features. Selection of features is performed randomly with replacement. In Step 2 the clustering process is performed on the training set. Consequently, *P_k_* number of clusters is generated according to the selected subset of features. The clustering process is performed by the *k*-means (weka.clusterers.SimpleKMeans) algorithm implemented in Weka [32] that uses the Euclidean distance measure (weka.core.EuclideanDistance). For each clustering process the number of clusters to be generated was randomly selected with the lower-bound equal to the number of classes in the classification problem being considered. In an effort to avoid empty clusters being generated, the upper bound was set to three times the number of classes. For each cluster obtained in the clustering process its centroid (weka.clusterers.SimpleKMeans.GetClustersCentoids) is identified. Each categorical/numerical feature of the centroid is calculated as a mode/average of the values of the features stemming from all instances within the cluster. It is assumed that each cluster supports one or more classes. For example, we say that a cluster provides a degree of support to class *c* if it contains at least one instance that belongs to this class. The level of support allocated for each class is dependent on the number of instances from the class and the total size of the cluster. In Step 3 a matrix *A_k_*, referred to as a support matrix, is constructed. This matrix represents the support provided to each class by each of the clusters in the collection. Each row in the matrix represents one cluster and each column represents one class. The value in cell *A*[*i*, *j*] presents the level of support provided to class *j* from cluster *i* and it is calculated as presented in Equation (1):

(1) $$A_{k}[i,j]=\{\begin{array}{ccc} \frac{N_{\text{ij}}-\overset{N_{i}}{\;}/\underset{M}{\;}}{N_{i}-\overset{N_{i}}{\;}/\underset{M}{\;}} & \text{if} & N_{\text{ij}}-\overset{N_{i}}{\;}/\underset{M}{\;}\geq 0\\ \frac{N_{\text{ij}}-\overset{N_{i}}{\;}/\underset{M}{\;}}{\overset{N_{i}}{\;}/\underset{M}{\;}} & \text{if} & \text{otherwise} \end{array}$$

where *N_i_* represents the total number of instances in cluster *i*, *N_ij_* refers to the number of instances from class *j* that belong to cluster *i*, and *M* stands for the number of classes in the classification problem being considered. Given that the clustering process is performed based on a randomly selected subset of features, it should not be expected that the majority of the instances within each cluster belong to the same class. Consequently, it is assumed that in some of the cases one cluster contains a mixture of instances from different classes. Based on this assumption, the value *N_i_*/*M* in Equation (1) represents an average number of instances from each class within cluster *i*. This step ensures that support provided for a class from one cluster is dependent on all instances within this cluster, not only instances from the class being considered. Consequently, the level of support is represented by values from the range [−1, 1]. This is considered as being beneficial while combining support coming from different clusters by application of an exponential function. A maximum level of support equal to 1 is obtained if all instances within the cluster belong to the same class. If for some classes there are no instances in the cluster, the support will be equal to −1. Based on this approach a value within the range [−1, 1] is assigned to a cell *A*[*i*, *j*] and subsequently represents the level of support given to class *j* from cluster *i*. As a result of the 3 step process we obtain one base classifier that is represented by the support matrix and the clusters' centroids. It is hypothesised that by taking into consideration support given for each class by a neighbourhood, rather than selecting just one class for each neighbourhood, a more accurate decision can be provided. The entire process is repeated *K* times, where *K* refers to the size of the ensemble required.

### 3.2. Combining Base Classifiers Outputs

In the classification process, following the presentation of a new instance, the closest cluster from each collection is selected. The selection is performed based on the distance between the centroid of the cluster and the new instance. Each of the selected clusters is represented by one row of the matrix. In the next phase of the classification process the level of support is calculated for each class based on the values from the selected rows and the distance between the centroids and the unseen instance, as presented in Equation (2):

(2) $$\text{ExSupp}(c_{j})=\sum_{k=1}^{k}\{\begin{array}{ll} e^{\frac{A_{k}[i_{k},j]}{1+d(x,x^{k})}} & \text{if}\;A_{k}[i_{k},j]>-1\\ 0 & \text{otherwise} \end{array}$$

where *i_k_* is the row from matrix *A_k_* representing the selected cluster and *d* represents the Euclidean distance metric. The class that obtained the highest support is considered as the final decision.

The value of the exponential function increases faster as the argument increases. Taking this into consideration, the level of support for a class will increase faster as the number of instances from this class in the cluster increases. We can notice from Equation (1) that while calculating the final support for each class, the support provided by all base classifiers in addition to the distance *d* between the new unseen instance and the centroid of the cluster are taken into consideration. In this way we can differentiate between the contributions being made from the selected clusters to the final decision. For classes with positive support the smaller the distance between the instance and the centroid, the larger the impact the cluster has on the final decision for this class. If the support given by a base classifier for a class is negative, then the effect is opposite. If the value in the matrix is −1 this can be interpreted as there being no support from the cluster for that given class.

## 4. Parameters Selection

With the presented approach two parameters, namely ensemble size (*K*) and the upper bound for the number of clusters, need to be selected before the final ensemble is generated. Beside this, the subset of features (*F_k_*) applied in the clustering process and the number of clusters (*P_k_*) need to be determined for each of the base classifiers. In an effort to minimise the over-fitting problem random selection of the variables *P_k_* and *F_k_* were performed as opposed to opting for a tuning process. Both parameters need to differ for all base classifiers in an effort to generate diversity within the ensemble. Tuning parameters for each base classifier will cause an over-fitting problem and will increase the time complexity. Randomisation has been presented in many studies as a successful manner of selecting parameters while generating a classifier ensemble [33,34]. It can be expected that random selection of the two attributes may affect the performance of the final ensemble. This is caused by the fact that each base classifier depends on the selected values of the two attributes. Consequently, for a small size of ensemble, it may be the case that a different model is obtained each time the training process is performed. One possible solution to alleviate this issue is the selection of an appropriately large size of ensemble. A number of experiments were performed in order to identify what size of the ensemble was required to minimise the standard deviation of the final accuracy. Six ensemble sizes were investigated. For each of them the training process was repeated 50 times and then the standard deviation of all the results was calculated. Based on the values presented in Table 1 we selected *K* = 30 as the size of the ensemble.

The upper bound was applied for *P_k_* in order to avoid large numbers of empty clusters being generated in the clustering process. We can expect that for low number of features selected f or clustering and for high *P_k_* values there is a great possibility for obtaining empty clusters. A number of experiments were performed varying the value of *P_k_*. It was found that after a certain point, only the number of empty clusters changes as a result of increasing the value of *P_k_*.

Consequently, we established the upper bound for *P_k_* equal to three times the number of the classes. Given that the selected value is linked with the training data set it is likely to cause an over-fitting problem. In order to obtain an optimal solution, the selection process needs to be performed for each new training data set. This can be considered as a limitation of the approach. It is anticipated that the number of clusters for each base classifier should be determined based on the subset of features selected for clustering and the training sample size. This will solve the problem of empty clusters and consequently no bound for *P_k_* will be required.

## 5. Complexity of the Method

The training process of CBCE can be viewed as being complex as it requires clustering the training set *K* number of times. The computational cost of the generation of one base classifier with the CBCE can be considered as a *k*-Means clustering process and can be estimated using the big-O notation as presented in Equation (3):

(3) O(i×P×N×l)

where *i* is the number iterations in the clustering process, *P* stands for the number of clusters, *N* represent the size of the training set and *l* is the number of features. Given that values *P* and *l* are selected randomly, the complexity time may vary for different iterations. It can be noted that for large data sets this process may be time consuming. For a single *k*NN classifier, applied as a benchmark in our study, no training is required. As an advantage of CBCE we can, however, consider it as being a computationally light classification process. A new instance needs to be compared only with the number of instances representing centroids. For one base classifier the computational complexity of the classification process can be estimated as:

(4) O(P×l)

Given the linear complexity the algorithm is still computationally attractive even if the number of features (sensors) is substantially large. For the single *k*NN, on the other hand, the classification complexity can be estimated as:

(5) O(N×l)

We can therefore note that for large data sets the process of classifying new instance is less complex for CBCE than for the *k*NN. With CBCE the size of the training set does not affect the classification time. Beside this, given that only clusters' centroids are applied in the classification process, there is no need to store all the data in the memory. Based on this, we can assume that CBCE is more suitable for real-time applications than *k*NN. In our previous work [6] we evaluated the complexity of CBCE in comparison to *k*NN. 20 data sets of different size were applied. Based on the experimental results we found that it takes more time to train the classifier ensemble, however, the CBCE is significantly more efficient than a single *k*NN in term of classification time.

## 6. Data Pre-Processing

In order to evaluate the CBCE in activity recognition we considered a well-known and publicly available data set [10]. All information regarding the data set, such as environment, sensors used and annotation applied during the data collection process can be found in [10]. In summary, sensor data were collected over a period of 26 days in a 3-room apartment from a 26 year old male subject. Fourteen wireless sensors were installed in the apartment, each associated with one object. The objects used in the experiments are presented in Table 2. Seven activities, as presented in Table 3, were observed throughout the duration of the experiments. In total there were 245 instances (activities) represented by 1230 sensor events.

The task, from an activity recognition perspective, is to classify an instance represented by a combination of sensor activations as belonging to one of the possible set of activities. For example:

[Hall‐Bathroom door,Toilet Flush,Toilet Flush,Hall‐Bathroom door]→use toilet

This can also be written as:

$$[S_{6},S_{14},S_{14},S_{6}]\to A_{4}$$

where *S_i_* is a sensor and *A_i_* is a class. CBCE is an instance-based method that applies the Euclidean distance metric to calculate the distance between two instances. For this reason data to be used in the current study should be represented as vectors with the same dimension. In the activity recognition problem being considered instances are represented as a sequence of numbers/strings that may have different lengths. To apply the previously developed CBCE methods with this data, the sensor recordings need to initially be converted into vectors of the same dimension. For this purpose each instance (sequence of sensors labels) was converted into a 14-dimensional vector. Each dimension of the vector represents one sensor (refer to Table 2):

$$\left[S_{1},S_{5},S_{6},S_{7},S_{8},S_{9},S_{12},S_{13},S_{14},S_{17},S_{18},S_{20},S_{23},S_{24}\right]$$

## 7. Numeric and Binary Data Representation

In the experiments, numeric and binary representations of the sensor recordings have been considered. For the numeric representation the position and value in the vector is an indicator of how many times the sensor appears in the sequence. For the binary representation, the value for each position is either 1 or 0 subsequently indicating if the sensor appears or does not appear in the sequence, respectively. As an example the activity *[Hall-Bathroom door, Toilet Flush, Toilet Flush, Toilet Flush, Hall-Bathroom door]* in the numeric system will be presented as:

[0,0,2,0,0,0,0,0,3,0,0,0,0,0]

We can read from this vector that sensors S_6_ (*Hall-Bathroom door*) and S_14_ (*Toilet Flush*) appeared in the sequence two and three times, respectively. The same activity in the binary system is represented as:

[0,0,1,0,0,0,0,0,1,0,0,0,0,0]

From the binary vector we can read that sensors S_6_ and S_14_ appeared in the sequence although we do not have any information relating to their number of occurrences. The two ensemble methods were evaluated with the data set represented by numeric and binary vectors.

## 8. Case Study

To help illustrate how the classifier ensemble is generated with the CBCE approach we present an exemplary case of the training process with the binary data set being considered. For the purposes of providing a simple example we select the size of the ensemble as 3. Consequently, the clustering process is performed three times with a randomly selected numbers of features and clusters. Table 4 presents the output of the training process. In the second column we can observe the centroids of the generated clusters. The third column shows home many instances from each class belong to the clusters. The number refers to the class labels in the following order: “*go-to-bed*”, “*use-toilet*”, “*prepare-breakfast*”, “*take-shower*”, “*get-drink*”, “*prepare-dinner*”, “*leave-house*”. Consequently, we can notice, that cluster number 1 from the first collection contains 14, 17 and 2 instances from classes “*go-to-bed*”, “*take-shower*” and “*prepare-dinner*”, respectively. The last columns present the support matrix calculated for each cluster collection according to Equation (1).

To help understand the decision process while classifying a new instance with CBCE we present an exemplary classification case followed by its analysis. Let us consider a classification process of the following instance: [0,0,0,0,0,1,0,0,0,1,0,0,0,0]. In the first step, the distance between a new instance and all the centroids is calculated. Following this, the closest cluster is selected from each collection. In the presented example clusters number 1, 8 and 5 were selected from the 3 collections based on the calculated distances:

$$\begin{array}{l} d\left(\left[0,0,0,0,0,\text{1},0,0,0,\text{1},0,0,0,0\right],\left[0,\text{1},0,0,0,0,0,0,0,0,0,0,0,0\right]\right)=1.7\\ d\left(\left[0,0,0,0,0,\text{1},0,0,0,\text{1},0,0,0,0\right],\left[0,0,0,0,0,\text{1},0,0,0,\text{1},0,0,\text{1},0\right]\right)=1\\ d\left(\left[0,0,0,0,0,\text{1},0,0,0,\text{1},0,0,0,0\right],\left[\text{1},0,0,0,0,\text{1},0,0,0,\text{1},0,0,0,0\right]\right)=1 \end{array}$$

In the next step, rows representing the selected cluster in the support matrix are selected:

$$\left[\begin{array}{lllllll} 0.3 & - & - & 0.4 & - & -0.6 & -\\ - & - & 0.4 & - & - & 0.4 & -\\ - & - & - & - & - & 1 & - \end{array}\right]$$

Following this, Equation (2) is applied in order to calculate the final support for each of the classes:

$$\begin{array}{c} \text{ExSupp}(\text{go}-\text{to}-\text{bed})=e^{0.3/(1+1.7)}=1.12\\ \text{ExSupp}(\text{use}-\text{toilet})=0\\ \text{ExSupp}(\text{prepare}-\text{breakfast})=e^{0.4/(1+1)}=1.22\\ \text{ExSupp}(\text{take}-\text{shower})=e^{0.4/(1+1.7)}=1.16\\ \text{ExSupp}(\text{get}-\text{drink})=0\\ \text{ExSupp}(\text{prepare}-\text{dinner})=e^{-0.6/(1+1.7)}+e^{0.4/(1+1)}+e^{1/(1+1)}=3.6\\ \text{ExSupp}(\text{leave}-\text{house})=0 \end{array}$$

We can observe that the highest value was obtained for the class “*prepare-dinner*”. Consequently, this class is considered as a final decision for the new activity.

## 9. Results and Discussion

The purpose of this study was to evaluate the performance of the CBCE ensemble method within the domain of activity recognition. Two main issues were considered, namely the performance of CBCE and the two types of activity representation. For the evaluation, we performed a 5-fold cross validation applying all the instances from the data set being considered. The process was performed five times. In each iteration four folds were applied for training and one fold was used for testing. The final accuracy was calculated as an average over the five results. In addition to accuracy, all the methods were evaluated using precision, recall and F-measure [35] (Table 5).

The columns and rows in the matrix refer to the actual and predicted classes by a classification model, respectively. Based on the confusion matrix the precision, recall and F-Measure for class 1 can be calculated as presented in Equations (6)–(8), respectively:

(6) $$\text{Precision}=\frac{TP_{11}}{TP_{11}+FP_{12}}$$

(7) $$\text{Recall}=\frac{TP_{11}}{TP_{11}+FN_{12}}$$

(8) $$\text{F}-\text{Measure}=\frac{2*\text{precision}*\text{recall}}{\text{precision}+\text{recall}}$$

The three measures were calculated for each class and the final results are presented as an average over all classes. These particular measures were used given that in real world applications there are issues associated with unbalanced data resulting in some classes appearing more often than others. In this way we can verify if the methods perform equally well for each class.

### 9.1. Classification Accuracy

**Figure 1.**
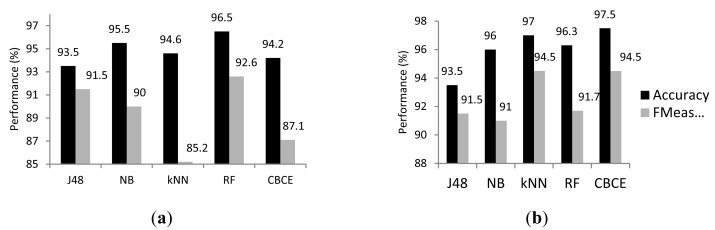
(**a**) Percentage value of accuracy and F-Measure obtained for sensor data with numeric representation. J48—J48 Tree, NB—Naive Bayes, kNN—K Nearest Neighbour, RF—Random Forrest, CBCE-Cluster-Based Classifier Ensemble; (**b**) Percentage value of accuracy and F-Measure obtained for sensor data with binary representation. J48—J48 Tree, NB—Naïve Bayes, kNN—K Nearest Neighbour, Random Forrest, CBCE—Cluster-Based Classifier Ensemble.

Three commonly applied single classification models were considered as a benchmark for the CBCE. The three algorithms, namely Naive Bayes (weka.classifiers.bayes.NaiveBayes) (NB), J48 Tree (weka.classifiers.J48 −C 0.25 −M 2) (J48) and *k* Nearest Neighbour (weka.classifiers. lazy.IBk −K 1 −W 0 −X −A) (*k*NN), were implemented in Weka. Beside the single classification models we compared CBCE against another classifier ensemble, namely Random Forest (weka. classifiers.trees.RandomForest −I 10 −K 0 −S 1 −num-slots 1) introduced earlier in Section 2.2. The random feature selection was performed for RF in the same manner as for CBCE. Results obtained by all the methods for numeric and binary data are presented in Figure 1a,b, respectively.

In an effort to gain a better insight into the activity recognition problem being considered, a confusion matrix was constructed. Tables 6 and 7 present the results obtained by the CBCE method applied with numeric and binary data, respectively.

Columns in both tables refer to actual classes of the instances from the testing set. Rows represent the class labels predicted by the CBCE method. The diagonal in each matrix represents the true positives indicating the instances which were correctly classified by the method. The sum of each column provides the total number of instances belonging to each class. The sum of each row provides the total number of instances which have been assigned to each particular class. The sum of the values in the diagonal divided by the sum of all of the values in the matrix provides the overall accuracy of the model.

We can observe from Figure 1 that the best accuracy was obtained by the two instance-based methods, namely CBCE (97.5%) and *k*NN (97.0%) with binary data. Both of the methods outperformed NB (96%), J48 (93.5%) and RF (96.3) in terms of accuracy and F-measure. This suggests that instance-based approaches are effective while applied in activity recognition problems. Besides the high general accuracy obtained by the CBCE we can observe, based on the precision and recall measures that the method performed well for each of the classes separately. From Table 6 we can notice that the ensemble method did not misclassify any of the instances from classes “*Leave house*”, “*Go to bed*” and “*Use toilet*”. This is probably due to the fact that activities from the first two classes are represented by different subsets of sensors than activities from any other class, which is what makes them easier to recognise. “*Use toilet*”, on the other hand, is the most frequent class hence instances from this class should be the easiest to classify. We can observe from Table 6 that for each class CBCE managed to correctly recognise between 80% and 100% of the instances. The most common mistake was confusing the two activities “*Prepare breakfast*” with “*Prepare dinner*”. The reason for this can be related to the fact that a large number of actions (sensors) are common to both of the activities.

In an effort to provide more insight into the CBCE generation process we investigated the performance of the base classifiers (BC) individually. Following each clustering process, we performed classification of the testing data in the same manner as with CBCE, however, applying only one base classifier. Based on the results obtained, we found that the most accurate single classifier was generated as the output of clustering process performed with all 14 features. We can infer from this that the *k*-Means classifier provides a better cluster collection than a random clustering process (with random subset of attributes). The comparison between CBCE and the best single classifier is presented in Table 8.

We can find from Table 8 that the *k*-Means obtained accuracy 95.3% which makes it more accurate than J48. It did not, however, outperform CBCE in terms of accuracy and it obtained significantly worse results in terms of Precision and Recall.

### 9.2. Features Representation

It can be observed from Figure 1 that for the CBCE and *k*NN, optimal results were obtained when the data was represented with the proposed binary system rather than the numeric system, however, for the single classifiers the difference in accuracy was marginal. Whilst applied with numeric data, the CBCE method obtained an accuracy level of 94.6%. Considering the approach with binary data the results were improved to 97.5% and the difference in accuracy was statistically significant (*p* = 0.003).

From Tables 6 and 7 it can be observed that the CBCE method performed worse in terms of classification accuracy while applied with numeric data than when it was applied with binary activity representation. We can infer from this that, whilst calculating the similarity between the two activities, it is more important to know which actions have been performed rather than how many times each actions took place. This can be explained by the fact that in the classification problem being considered the same activity can be represented by different combinations of actions. For example, while cooking dinner the fridge may be opened a different number of times. This may cause some problems while calculating the Euclidean distance between the same activities that have been performed in two different ways. It may also appear that two instances from the same class will be considered as being very distant. If we compare Table 6 with Table 7 we can notice, for example, that activities “*Prepare dinner*” is confused with “*Prepare breakfast*” more often when presented in numeric rather than the binary system. Based on the instances from the data set we can see that activity “*Prepare breakfast*” is mainly composed of actions related with sensors “*Fridge*”, “*Plates-cupboard*” and “*Groceries-cupboard*”. For activity “*Prepare dinner*” some other sensors like “*Freezer*” or “*Pans-cupboard*” are involved. As an example let us consider 3 instances represented in the numeric system taken from the data set:

$$\begin{array}{c} I_{1}=[0,0,0,0,\text{2},\text{2},0,0,0,0,0,0,\text{3},0{,}^{\prime }\text{prepare}-{\text{breakfast}}^{\prime }]\\ I_{2}=[\text{0},\text{0},\text{0},\text{0},\text{3},\text{3},\text{0},\text{0},\text{0},\text{1},\text{2},\text{0},\text{1},\text{0}{,}^{\prime }\text{prepare}-{\text{dinner}}^{\prime }]\\ I_{3}=[\text{0},\text{0},\text{0},\text{0},\text{3},\text{2},\text{0},\text{0},\text{0},\text{4},\text{0},\text{0},\text{2},\text{0}{,}^{\prime }\text{prepare}-{\text{dinner}}^{\prime }] \end{array}$$

If we calculate the Euclidean distance between each example we obtain: $d(I_{1},I_{2})=\sqrt{11}$, $d(I_{2},I_{3})=\sqrt{15}$ and $d(I_{1},I_{3})=\sqrt{18}$. Based on these calculations instances *I_1_* and *I_2_* have a greater chance to be located in the same cluster, rather than *I_2_* and *I_3_*, even though they represent different activities. If the instances were presented in the binary system, then we would obtain: $d(I_{1},I_{2})=\sqrt{2}$, *d*(*I*_2_, *I*_3_) = 1, *d*(*I*_1_, *I*_3_) = 1. In this case, the probability of locating instances from two different classes in the same cluster is smaller than in the first case.

From the example presented above we can observe that the main problem for the numeric representation of data is related with the fact that some activities may be performed in many different ways. Binary representation of activities, even though it provides less information, seems to be more appropriate when applied with instance-based classification models. An additional experiment was conducted in an effort to investigate the difference between numeric and binary data representations in more detail. All instances from the data set were divided into seven clusters according to the activity they represent. Following this, a clusters' evaluation metric was applied for both, binary and numeric, cluster collections. Davies-Boudlin Index was applied [36], given that it considers both, internal and external, similarity of the instances from different clusters. The smaller value of the DB Index, the better the quality of the clusters. Table 9 presents values of the DB Index for both, numeric and binary, activity representation. Based on the results we can presume that the activities from the data set being considered are better defined with binary representation. This may justify why CBCE performed better with this type of data.

It can be noted that by representing activities as binary vectors the information related to the order that the sensors are activated and the number of occurrences is lost. Consequently, it may be the case that for some data sets, with more complex activities, the binary representation will not be the optimal solution. This may happen if two activities are represented by the same set of sensors, however, differ with the number and order of sensor activations. Beside this, information relating to the order and the number of occurrences may be significant while trying to recognise abnormal human behaviours. In the next step of this study we wish to evaluate CBCE with data collected from various environments and investigate different activity representations in more detail. In future work, we aim to extend the approach in the manner that will allow the recognition of anomalous activities. For this purpose, we will consider feature representation that provides more information rather than sensor activations only.

## 10. Conclusions and Future Work

This study provides a basis for further investigation into the application of ensemble methods in activity recognition within the application domain of smart homes. The CBCE ensemble method has been evaluated with an activity recognition problem and it was presented to be more accurate than a number of single classifiers. All the classification methods considered in this study were applied with data represented in both a numeric and binary format. It has been demonstrated that the method performed better when applied to binary data. In addition, it can be concluded that instance-based methods are beneficial when applied in activity recognition problems compared to other classification techniques. Apart from high accuracy it was demonstrated that CBCE is computationally light in terms of its classification process. Beside this, in our previous study it was presented that CBCE deals much better with a small size of training set compared with a number of different single classifiers. Following this analysis, we can infer that the proposed approach can be considered for a real world application, such as activity recognition in smart homes.

The study presented in the paper may be considered a preliminary analysis and further work is still intended. The first problem to be considered in the future work is an improved approach to selecting parameters for the model. It is presumed that the appropriate selection of the number of clusters and the subset of features applied in the clustering process will improve the performance of the model.

In our future work we wish to investigate in further detail the two types of activity representations and their relations with the performance of the model. It was presented earlier in the paper that instance-based classification methods perform better with binary rather than numeric representation of activities. We wish to consider this issue in more detail, taking into consideration situations when the order and number of occurrences may be significant, such as recognising anomalous behaviours or more complex activities.

## Figures and Tables

**Table 1.** Standard deviation of accuracy obtained by CBCE for different numbers of base classifiers (K).

**Ensemble Size**	**1**	**10**	**20**	**30**	**40**	**50**
St. Dev.	15.34	3.5	1.7	0.47	0.42	0.41

**Table 2.** List of the fourteen sensors installed in the apartment (van Kasteren, [10]).

**Sensor ID**	**Sensor Label**
1-	‘Microwave’
5-	‘Hall-Toilet door’
6-	‘Hall-Bathroom door’
7-	‘Cups cupboard’
8-	‘Fridge’
9-	‘Plates cupboard’
12-	‘Front door’
13-	‘Dishwasher’
14-	‘Toilet Flush’
17-	‘Freezer’
18-	‘Pans Cupboard’
20-	‘Washing machine’
23-	‘Groceries Cupboard’
24-	‘Hall-Bedroom door’

**Table 3.** List of activities observed in experimental data set (van Kasteren, [20]).

**Activity ID**	**Activity Label**	**No. of Activities**
1-	‘Leave house’	34
4-	‘Use toilet’	112
5-	‘Take shower’	23
10-	‘Go to bed’	24
13-	‘Prepare breakfast’	20
15-	‘Prepare dinner’	10
17-	‘Get drink’	22

**Table 4.** Output of the training process of the CBCE for the input parameter “*ensemble size*” equals 3.

	**Step 1**	**Step 3**	

	**Centroids**	**Number of Instances in Each Cluster**	**Support Matrix**
1	**[0,1,0,0,0,0,0,0,0,0,0,0,0,0]**	**14 0 0 17 0 2 0**	**0.3 −1 −1 0.4 −1 −0.6 −1**
2	[0,0,0,0,0,0,1,0,0,0,0,0,0,0]	0 0 0 0 0 0 25	−1 −1 −1 −1 −1 −1 1
3	[0,0,1,0,0,0,0,0,1,0,0,0,0,0]	0 69 0 0 0 0 0	−1 1 −1 −1 −1 −1 −1
4	[0,0,0,1,1,0,0,0,0,0,0,0,0,0]	0 0 1 0 12 0 0	−1 −1 −0.5 −1 0.9 −1 −1
5	[0,0,0,0,1,1,0,0,0,0,0,0,1,0]	0 0 16 0 2 0 0	−1 −1 0.9 −1 −0.2 −1 −1
6	[0,0,0,1,0,0,0,0,0,0,0,0,0,0]	0 0 0 0 1 0 0	−1 −1 −1 −1 1 −1 −1
7	[0,0,0,0,0,0,0,0,1,0,0,0,0,0]	0 13 0 0 0 0 0	−1 1 −1 −1 −1 −1 −1
8	[0,0,1,0,0,0,0,0,0,0,0,0,0,0]	0 10 0 1 0 0 0	−1 0.9 −1 −0.4 −1 −1 −1
9	[1,0,0,1,1,1,0,0,0,1,1,0,1,0]	0 0 1 0 0 3 0	−1 −1 0.1 −1 −1 0.7 −1
10	[0,0,0,1,0,1,0,1,0,0,1,0,1,0]	0 0 0 0 0 1 0	−1 −1 −1 −1 −1 1 −1
11	[0,0,0,1,1,0,0,1,0,1,0,0,0,0]	0 0 0 0 1 0 0	−1 −1 −1 −1 1 −1 −1
12	[0,0,0,0,1,1,0,0,0,1,1,0,1,0]	0 0 0 0 0 2 0	−1 −1 −1 −1 −1 1 −1
13	[0,0,0,0,1,1,0,1,0,1,1,0,0,0]	0 0 1 0 0 0 0	−1 −1 1 −1 −1 −1 −1

1	[0,0,0,0,0,0,0,0,0,0,0,0,0,1]	14 0 0 0 0 0 0	1 −1 −1 −1 −1 −1 −1
2	[0,1,0,0,0,0,0,0,0,0,0,0,0,0]	0 2 1 18 0 0 0	−1 −0.3 −0.7 0.8 −1 −1 −1
3	[0,0,0,0,0,0,1,0,0,0,0,0,0,0]	0 0 0 0 0 0 24	−1 −1 −1 −1 −1 −1 1
4	[0,0,1,0,0,0,0,0,1,0,0,0,0,0]	0 74 0 0 0 0 0	−1 1 −1 −1 −1 −1 −1
5	[0,1,0,0,0,0,1,0,0,0,0,0,0,0]	0 0 0 0 0 0 1	−1 −1 −1 −1 −1 −1 1
6	[0,0,0,1,1,0,0,0,0,0,0,0,0,0]	0 0 2 0 13 3 0	−1 −1 −0.2 −1 0.7 0.03 −1
7	[0,1,1,0,0,0,0,0,1,0,0,0,0,0]	0 11 0 0 0 0 0	−1 1 −1 −1 −1 −1 −1
8	**[1,0,0,0,0,1,0,0,0,1,0,0,0,0]**	**0 0 2 0 0 2 0**	**−1 −1 0.4 −1 −1 0.4 −1**
9	[0,0,0,0,1,1,0,0,0,0,0,0,1,0]	0 0 14 0 2 2 0	−1 −1 0.7 −1 −0.2 −0.2 −1
10	[0,0,0,1,0,0,0,0,0,0,0,0,0,0]	0 0 0 0 1 1 0	−1 −1 −1 −1 0.4 0.4 −1
11	[0,1,0,0,0,0,0,0,1,0,0,0,0,0]	0 5 0 0 0 0 0	−1 1 −1 −1 −1 −1 −1

1	[0,0,0,0,0,0,0,0,0,0,0,0,0,1]	9 3 0 0 0 0 0	0.7 0.1 −1 −1 −1 −1 −1
2	[0,1,0,0,0,0,0,0,0,0,0,0,0,0]	0 18 0 18 0 0 1	−1 0.4 −1 0.4 −1 −1 −0.8
3	[0,0,1,0,0,0,0,0,1,0,0,0,0,0]	0 71 0 0 1 0 24	−1 0.7 −1 −1 −0.9 −1 0.1
4	[0,0,0,1,1,0,0,0,0,0,0,0,0,0]	0 0 1 0 15 0 0	−1 −1 −0.6 −1 0.9 −1 −1
5	**[0,0,0,0,0,1,0,0,0,1,0,0,1,0]**	**0 0 0 0 0 3 0**	**−1 −1 −1 −1 −1 1 −1**
6	[0,0,0,0,1,1,0,0,0,0,0,0,1,0]	0 0 18 0 0 5 0	−1 −1 0.8 −1 −1 0.1 −1
7	[0,1,0,0,0,0,0,0,0,0,0,0,0,1]	5 0 0 0 0 0 0	1 −1 −1 −1 −1 −1 −1

**Table 5.** Confusion matrix presenting number of true positives, true negatives, false positives and false negatives for a 2 class classification problem.

		**Actual Class**	

		**1**	**2**
Predicted Class	1	TP_11_ (true positive)	FP_12_ (false positive)
	2	FN_12_ (false negative)	TN_22_ (true negative)

**Table 6.** Confusion matrix for the CBCE method applied with numeric data.

	**Bed**	**Toilet**	**Breakfast**	**Shower**	**Drink**	**Dinner**	**Leave**
Bed	23						
Toilet		109					
Breakfast			18		1	8	
Shower		3		23			
Drink			1		19		
Dinner			1			2	
Leave							32

**Table 7.** Confusion matrix for the CBCE method applied with binary data.

	**Bed**	**Toilet**	**Breakfast**	**Shower**	**Drink**	**Dinner**	**Leave**
Bed	23						
Toilet		112		1			
Breakfast			17			2	
Shower				22			
Drink					20		
Dinner			3			8	
Leave							32

**Table 8.** Percentage results obtained for CBCE and the best base classifier (BC) applied with numeric and binary data.

**Classification Method**	**Data**	**Precision [%]**	**Recall [%]**	**F-Measure [%]**	**Accuracy [%]**
CBCE	Numeric	88.3	86.6	87.1	94.2
	Binary	94.3	94.5	94.4	97.5
Best BC (14 attributes)	Numeric	78.1	80.0	80.4	91.9
	Binary	87.8	90.5	89.0	95.3

**Table 9.** DB Index calculated for collection of activities in binary and numeric representations.

	**Binary Representation**	**Numerical Representation**
Davies Bouldin Index	1.03	1.6
